# Nest site availability and niche differentiation between two cavity‐nesting birds in time and space

**DOI:** 10.1002/ece3.5698

**Published:** 2019-09-27

**Authors:** Ping Ye, Canchao Yang, Wei Liang

**Affiliations:** ^1^ Ministry of Education Key Laboratory for Ecology of Tropical Islands College of Life Sciences Hainan Normal University Haikou China

**Keywords:** evolutionary partitioning, ghost of competition past theory, ghost of competition present theory, niche differentiation, niche separation

## Abstract

Niche differentiation is a key concept in the field of ecology and refers to the process by which competing species within an ecological community partition utilization of environmental resources to achieve coexistence. The existence of niche differentiation is uniquely difficult to prove on account of the fact that historical interaction among species, which plays a key role in elucidating the current state of coexistence among species, is not well known. We created continuous niche gradients in nest‐site resources between two sympatric secondary cavity‐nesting birds, the green‐backed tit (*Parus monticolus*) and the russet sparrow (*Passer cinnamomeus*), and investigated whether nesting site is a factor contributing to limiting breeding overlap by regular inspection and 388,160 min of film recording. Our results indicate that although we manipulated nest site availability to be uniformly high along the habitat gradient, the two bird species have little overlap in nest sites and rarely compete for them. Furthermore, the green‐backed tit possessed a wide range of fundamental niche that covered that of the russet sparrow, while their reproductive time was largely segregated. The sparrow was more aggressive and outcompeted the tit in their overlapped range. These results suggest that even though nesting sites are crucial to the reproduction of cavity‐nesting birds, some other factor plays a more important role in limiting niche overlap between sparrows and tits in space and time. Given that these two cavity‐nesting birds continued to use different habitats and breed in segregated time after our manipulation, their relationship is better explained by the ghost of competition past theory.

## Graphical abstract

To understand the process of niche differentiation, we created conditions for the overlapping formation of nest‐site resources between two cavity‐nesting birds. Their nest site selection is naturally partitioning, but after our manipulation, unequal competition was detected with one species outcompeting the other. The detected pattern of competition supports the ghost of competition present theory.

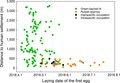

## 1 INTRODUCTION

Diversity, competition, and coexistence are among ecology's classical subjects. In an ecological community, various species live together as the end product of an assembly process by which organisms interact with each other. Some organisms positively interact (e.g., mutualistic and commensal behavior), while others negatively interact (e.g., predation, parasitism, and competition) (Hizel & Lay, 2008). Negative interaction gives rise to a question as to how the interacting members of different species persist together without one driving the other to extinction? Niche differentiation (or niche separation or niche partitioning), whereby coexisting and competing species use environments in different ways, was proposed to explain the interaction of competition (Miller, Terhorst, & Burns, 2009).

In the breeding season, animals may need greater environmental resources due to their own requirements and those of their offspring. Furthermore, nests are important sites for rearing of offspring. Therefore, animals may be sensitive to nest site overlap and competition during the breeding season. Most previous studies that have investigated nest site competition in animals have focused on mammals (Edelman, Koprowski, & Bertelsen, 2009; Schradin, 2005), birds (Goldshtein, Markman, Leshem, Puchinsky, & Charter, 2018; Quintana & Yorio, 1998; Trivelpiece, Trivelpiece, & Volkman, 1984; Weitzel, 1988), fishes (Breitburg, 1987; Kroon, de Graaf, & Liley, 2000; Saaristo, Craft, Lehtonen, & Lindstrom, 2009), and insects (Alcock, 1982; Cerda & Retana, 1998; Dooley & Dueser, 1996; Schneider, Deeby, Gilley, & DeGrandi‐Hoffman, 2004). For passerine birds, especially those species that are secondary cavity nesting, nest sites are crucial aspects of their life histories; competition for nest sites must be severe, because (a) such species build nests in cavities as places for incubating their eggs and rearing their offspring, and (b) they cannot excavate cavities by themselves, but instead search for ready‐made holes that are generally limiting resources in nature. Although previous studies have demonstrated some nest site competition within or among birds, most of those studies focused on the conservation issue due to nest competition between introduced and native species (Charter, Izhaki, Ben Mocha, & Kark, 2016; Heinsohn, Murphy, & Legge, 2003; Hernandez‐Brito, Carrete, Ibanez, Juste, & Tella, 2018; Ingold, 1994; Kerpez & Smith, 1990).Very few studies have investigated the effect of nest‐site competition on habitat overlap (Minot & Perrins, 1986).

In this study, we provided abundant artificial nesting sites with identical features along a habitat gradient for two cavity‐nesting birds and tested whether nest‐site competition was responsible for limiting habitat overlap between them. According to the recorded data and our own observations, these two studied species, the green‐backed tit (*Parus monticolus*) and the russet sparrow (*Passer cinnamomeus*), naturally breed sympatrically in our study area in southwest China with some overlapping in nest site requirements. The russet sparrow nests in holes in a tree, in‐house eaves or other building cavities, in embankments, or in stone walls, while the nest sites of the green‐backed tit cover a range from holes or cavities in tree trunks, old stumps, or fence posts, to holes in rocks, earth banks, holes in walls, or under house eaves (del Hoyo, Elliott, Sargatal, & Christie, 2013). This implies that the two species have similar requirement for nesting sites that they may compete for them after our manipulation. Despite similar nesting requirements, russet sparrows and green‐backed tits have different patterns of habitat occupation. Within the populations studied, all observable russet sparrows were found to be living and breeding in human settlements (Huo, Su, Niu, Yang, & Liang, 2018; Yang et al., 2012), while green‐backed tits were living from human settlements to forest areas, but their nests were rarely found in human settlements (Yang, Liang, & Møller, 2019). Competition for nesting sites of these two species should be under strong selection pressure because: (a) the nest site is an essential resource for avian reproduction that determines how many offspring or genes birds can transmit to the next generation; (b) green‐backed tit and russet sparrow are both secondary cavity‐nesting birds that cannot make, but can only occupy, existing cavities for breeding; (c) cavities are much more limited as a resource than other nest sites such as bushes or trees; and (d) unlike flexible resources such as food, competition for cavities is a typical example of interference competition in that cavities cannot be used by different pairs of birds at a time within most stages of the breeding cycle that last more than one month (i.e., stages including nest building, egg laying, egg incubation, and chick feeding). Our intention was to test whether the availability of nest sites, which is an important factor for the reproduction of cavity‐nesting birds, affects habitat niche overlap between the green‐backed tit and the russet sparrow. Although the two cavity‐nesting species show different patterns of habitat occupation in our study area, by setting up artificial nest boxes we made the cavity resources uniform along the habitat gradient from human settlement to forest and thus provided a situation to promote potential interaction in nest site utilization between them. This study thus allows us to investigate whether nesting site was a factor contributing to limiting breeding overlap and may also provide referential information to better understand the theories of niche differentiation.

## 2 MATERIALS AND METHODS

### 2.1 Study area and experimental procedure

The study was performed in the Kuankuoshui Nature Reserve (28°10′N, 107°10′E) located in Southwest China. The study area is situated in a subtropical moist broadleaf and mixed forest at an altitude of about 1,500 m, in which tea plantations and several buildings were established to create an open area (Yang et al., 2010, 2015). A total of 213 nest boxes 35 cm in depth and 4 cm in entrance diameter were set up at 3 m height on trees in areas ranging from human settlement to the forest during March before the initiation of the breeding season. The interval distances between nest boxes ranged from ca. 15 to 350 m according to the tree position, forming the distances from nest boxes to human settlement that ranged from 45.7 to 3,264.2 m (GPS: G128BD, UniStrong Inc.). We chose human settlement rather than forest edge as a reference setting from which to measure the distance of nest boxes because the habitat is characterized by human settlement surrounded by forests, so using human settlement as a reference is obviously more feasible and precise. We monitored all nest boxes twice weekly to inspect the circumstances of occupation by the russet sparrow or the green‐backed tit. The frequency of checking was later changed to every other day when nest materials were found in nest boxes and every day when nest cups were shaped. Fifteen video monitoring cameras with a total of 388,160 min of film recording (eight cameras and 230,296 min for eight tit nests and seven cameras and 157,864 min for seven sparrow nests) were mounted to collect behavioral pattern data and provide evidence of competition. In regards to intraspecific or interspecific competition for nest sites among these two species, one will peck, destroy, and remove the other's eggs, subsequently add new nest materials on the next day, and then lay its own eggs a couple of days later (Videos [Link], [Link], [Link]).

### 2.2 Statistical analyses

The chi‐square test was used for comparison of the rates of nest box occupation, Levene's test for equality of variances, and Welch's *t* tests were used for comparing variances and means of distances of occupied nest boxes to human settlements, respectively. Furthermore, Welch's *t* test was also used for comparing breeding period data. Statistical analyses were performed by using IBM SPSS 25.0 for Windows (IBM Inc.). All tests were two‐tailed, and data were presented as mean ± *SE*.

## 3 RESULTS

**Figure 1:**
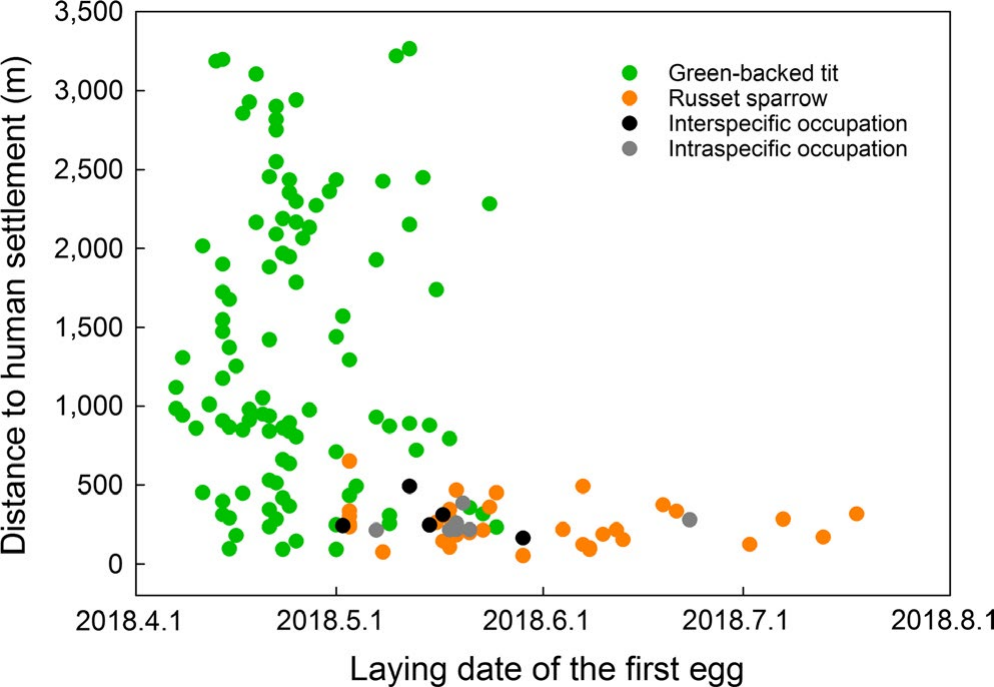
Overlap of nest box utilization in space and time between the green‐backed tit and russet sparrow. Nest boxes with interspecific occupation between tit and sparrow were displayed by dark circles. For all occupation cases, the russet sparrow displaced the green‐backed tit. Intraspecific occupation (gray circles) refers to the same nest boxes being occupied repeatedly by different individuals of the same species

The occupation rates of nest boxes in the green‐backed tit and the russet sparrow were 49.3% (105/213) and 21.1% (45/213,) respectively, which were significantly different (*χ* ^2^ = 37.04, *df* = 1, *p* < .001, Chi‐square test). The green‐backed tit and the russet sparrow overlapped in nest box utilization. The distance to human settlement of nest boxes occupied by tits ranged from 90.4 to 3,264.2 m, while for sparrows it ranged from 51 to 650.5 m. Therefore, the range of the green‐backed tit almost covered the entire range of the russet sparrow (Figure 1). The range of the green‐backed tit was wider (*F* = 108.9, *p* < .001, Levene's test for equality of variances) and further from human settlement (tit: 1,345.7 ± 88.5 m; sparrow: 250.7 ± 17.6 m; *t* = 12.14, *df* = 112.03, *p* < .001, Welch's *t* test) than the russet sparrow. In the overlapped range of nest boxes, five green‐backed tit nests were finally occupied by russet sparrows, with an occupation rate of 4.8% against the total number of tit nests (5/105). However, if we only consider the number of tit nests that overlapped with sparrow nests (i.e., were within the range of sparrow nests), the occupation rate increases significantly from 4.8% to 17.9% (*χ* ^2^ = 5.5, *df* = 1, *p* = .034, chi‐square test).

For the five tit nests occupied by sparrows, two were initially occupied by sparrows when empty; tits subsequently occupied sparrow nests during the sparrow nest building stage and began laying eggs, but these two nests were finally counter‐occupied by sparrows, with one occupied during tit egg laying stage and one during egg incubation stage. The other three tit nests were directly occupied by sparrows, with one occupied during tit nest building stage and two during egg laying stage. Both male and female sparrows participated in nest occupation. The sparrows laid eggs in four out of five occupied nests while one was occupied by adding nest materials without laying eggs. The nest materials of the green‐backed tit and the russet sparrow obviously were different in that they used moss and dry grass, respectively. When occupation occurred in the nest building stage, one added its own nest materials onto those of the other and then laid eggs after the nest was built. When occupation occurred in either the egg laying or the egg incubation stage, one pecked, destroyed, and removed the other's eggs, began to add its own nest materials on the day after egg removal and then laid eggs after the new nest was built (in one case of occupation only nest materials were added, see above). In addition to these five cases of interspecific occupation, seven cases of intraspecific occupation (hereafter meaning that the same nest boxes were occupied repeatedly by different individuals of the same species) were also detected with the russet sparrows, with one occurring during the egg laying stage and six during the egg incubation stage. In addition, four out of seven cases were associated with destruction of the eggs and nest materials without egg laying. However, no intraspecific occupation was found in the green‐backed tits.

**Figure 2:**
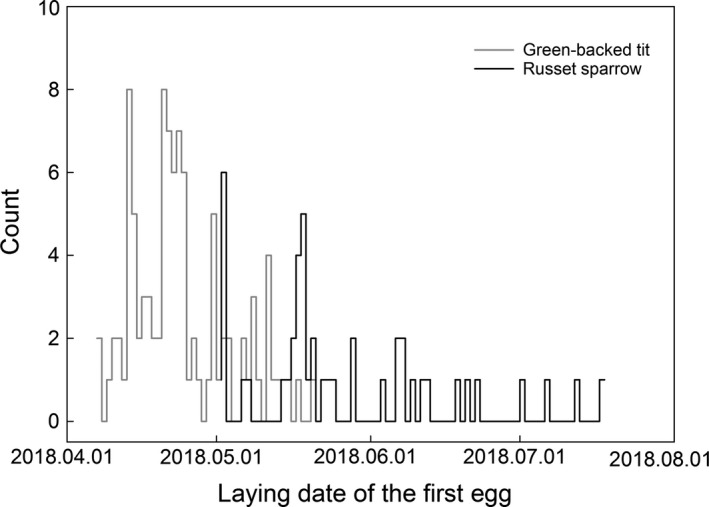
Reproductive time of the green‐backed tit and russet sparrow as represented by laying date of the first eggs

Although the green‐backed tit and the russet sparrow overlapped in some nest box utilization after our manipulation, their reproductive timing was significantly different. The first egg laying date of green‐backed tits ranged from 7 April to 25 May, while for russet sparrows it ranged from 2 May to 18 July (Figure 1). On that account, the green‐backed tit reproduced much earlier than the russet sparrow (*t* = 2.8, *df* = 133.06, *p* = .006, Welch's *t* test). Furthermore, the reproductive peak of green‐backed tits occurred outside the reproductive time of russet sparrows (Figure 2). Therefore, there is one nest box that was used by both green‐backed tits and russet sparrows without competition because the interval of occupation time between them was a month and a half (first egg laying date: 23 April and 8 June for tits and sparrows, respectively).

## 4 DISCUSSION

In our studied population, green‐backed tits and russet sparrows had little to no observed overlap in breeding habitat use before our manipulation of nest sites, with russet sparrows primarily nesting in human settlement and green‐backed tits in surround forest. By creating uniform nesting resources along a gradient of distance from human settlement, we tested whether competition of nest sites was responsible for limiting habitat overlap between these species or whether this instead reflects differences in their fundamental niche. Our results indicated that green‐backed tits possessed a wider range of nest box utilization than russet sparrows. Russet sparrows only used nest boxes that were close to human settlement, while green‐backed tits used nest boxes in an area from human settlement to forest. Therefore, their utilization of nest boxes overlapped in the range close to human settlement. Competition and interaction were observed between them; green‐backed tits occupied two russet sparrow nests during nest building stage, while sparrows occupied one tit nest during nest building stage and one during egg laying stage. Nevertheless, russet sparrows eventually outcompeted green‐backed tits and excluded them from these nest boxes.

Choosing living or breeding sites close to human settlement is an adaptive way to reduce predation risk because many predators keep far away from human beings (Liang, Yang, Wang, & Møller, 2013; Møller, 2010). Several sparrow species have adapted to live and breed in human settlements, including the russet sparrow (del Hoyo et al., 2013). Our results suggest that the russet sparrow is a better competitor that has specialized in human settlement, while the green‐backed tit is inferior to the sparrow in competition but is generalized to use a wide range of nesting sites. It has classically been proposed that generalists bear a cost that will reduce their fitness relative to a specialist using the same resource (Buchi & Vuilleumier, 2014; McPeek, 1996; Morris, 1996). In the case studied, the green‐backed tit is analogous to the generalist that is outcompeted and excluded by the specialist russet sparrow. In line with the ghost of competition present theory, generalists such as the green‐backed tit possess a wide fundamental niche but submit to specialists such as the russet sparrow if their niches are overlapped. The realized niche of the green‐backed tit we observe in natural circumstances thus differentiates from the russet sparrow by avoiding cavities near human settlement. Our study promoted overlap in nest site utilization between sparrows and tits because we provided abundant and uniform nest types along a gradient in distance from human settlement. Moreover, in addition to niche partitioning of cavities in space, time segregation is another important mechanism to achieve coexistence between green‐backed tits and russet sparrows.

A recent study found that climate change has led to breeding time overlap between great tits (*P. major*) and pied flycatchers (*Ficedula hypoleuca*). They are both secondary cavity‐nesting birds, and the competition between them led to a phenomenon in which great tits killed pied flycatchers (Samplonius & Both, 2019). Our study found that most of the reproductive time was separated between green‐backed tits and russet sparrows. Green‐backed tits reproduced earlier than russet sparrows, and their reproductive peaks did not overlap. Although such time segregation may not be a specific adaptation in tits to avoid competition with sparrows, it undoubtedly contributes to coexistence between them. Such time differentiation also plays an important role in their coexisting because it is not just related to nesting sites but is also related to other resources such as food. Green‐backed tits and russet sparrows are both residents, and in nonbreeding season, they utilize different food resource because green‐backed tits are insectivorous, while russet sparrows are herbivorous. However, during breeding season, they both feed insects to their nestlings. Although their nesting sites have partitioned, green‐backed tits also forage in human settlement. Therefore, time segregation is crucial for them to partition their food resources in breeding season. In short, the niche differentiation in space and time favors green‐backed tits and russet sparrows to avoid or reduce competition during breeding season.

Moreover, it is worth mentioning that intraspecific competition for nesting site was also detected in our results. For the russet sparrow, six nests were occupied by conspecifics of different individuals, accounting for 15.6% (*n* = 45 nests). However, intraspecific occupation was not detected in the green‐backed tit (*n* = 105 nests), even though its breeding population size was larger than that of the russet sparrow. This implies that russet sparrows are more aggressive than green‐backed tits for nest occupation. Aggressive behavior of this sort would significantly affect the outcome of competition (Miller et al., 2014; Pintor, Sih, & Bauer, 2008). As one can imagine, the better competitor in food resources depends on how good it is in finding and handling food, and thus, it outcompetes less competitive species indirectly. However, nest occupation represents direct contact and competition that more aggressive species can be expected to dominate over less aggressive species during interaction. The difference in aggression between green‐backed tits and russet sparrows possibly may be due to differences in body size (sparrow: 13–23 g, tit: 12–16.8 g) or personality.

In summary, although competition between sparrows and tits was confirmed after our manipulation, such competition cases were rare in consideration of the number of nest box we provided (5 competition out of 213 nest boxes). In other words, the habitat differences between these two species exist even though identical nest boxes were present along the habitat gradient. Furthermore, the breeding period of sparrows and tits was mostly separated that they continued to breed at different times even though there was an abundance of unutilized nest boxes. These indicate that although nesting site seems to be a crucial factor for reproduction of secondary cavity‐nesting birds, it is clearly not the only and important factor limiting overlap in habitat use and breeding times between russet sparrows and green‐backed tits. Some factor other than the type and availability of nest sites is responsible for limiting the distributions of these two species in space and time. First, the nest boxes provided a uniform nesting resource along the human settlement‐forest habitat gradient, indicating that any potential cryptic differences in nest sites between these habitats are evidently not the cause of spatial exclusion between the two species. Second, the nest boxes provided abundant and probably nonlimiting nesting sites, and thus competition for nest sites was also probably not the cause of nonoverlapping distributions in space and time between the species. Finally, although this study could not provide clear evidence to support anyone of the theories of niche differentiation, it is likely to be better explained by the ghost of competition past theory (Howe & Brown, 2001; Levin, 2006; Siemers & Schnitzler, 2004) because the breeding activities of the russet sparrows and green‐backed tits overlapped little in time and space when we provided abundant nesting site and gave opportunity for their competition.

Besides the ghost of competition past theory, the ghost of competition present theory (Connell, 1980; Miller et al., 2009) and the evolutionary partitioning theory (Hizel & Lay, 2008) have also been proposed to explain competition and niche differentiation. Ecologists have tried different ways to test these distinct hypotheses and find an explanation for niche differentiation. As mentioned above, however, proving niche differentiation is difficult because it is impossible to detect in action. Ecologists therefore generally have studied niche differentiation by either mathematical models or via present niche comparisons (Case & Gilpin, 1974; Dunbar & Dunbar, 1974; Grace & Wetzel, 1982; Jácomo, Silveira, & Diniz‐Filho, 2004; Loveridge & Macdonald, 2003; Peterson & Holt, 2003; Schmidt, Earnhardt, Brown, & Holt, 2000; Wasserberg, Kotler, Morris, & Abramsky, 2006; Zillio & Condit, 2007), although controlled experiments have occasionally been involved (Howe & Brown, 2001; Janssen, Van Alphen, Sabelis, & Bakker, 1995; Sack, 2004; Spina, 2000; Steiner, Cáceres, & Smith, 2007). In empirical studies, ecologists have tried to quantify the concepts of niche breadth, competition, and coexistence. But, many of these are distinctly difficult to measure quantitatively, especially in ecological studies. Because historical interactions among species and the process of niche differentiation are invisible to observers, we suggest that an optimal way to study niche differentiation may be by reconstructing niche overlap among species by empirical manipulation.

## CONFLICT OF INTEREST

The authors declare that they have no conflict of interest.

## AUTHOR CONTRIBUTION

C.Y. designed the study; C.Y. and P. Y. performed field experiments; C.Y. carried out laboratory and statistical analyses; C.Y. and P. Y. wrote the draft manuscript; and W. L. helped improve the manuscript. All authors approved the final submission.

## ETHICAL APPROVAL

The experiments reported here comply with the current laws of China. Fieldwork was carried out under the permission from Kuankuoshui National Nature Reserve, China. Experimental procedures were in agreement with the Animal Research Ethics Committee of Hainan Provincial Education Centre for Ecology and Environment, Hainan Normal University (permit nos. HNECEE‐2011‐001 and HNECEE‐2011‐002).

## Data Availability

Data used in this study are available in the electronic supplementary material (Data S1).
